# Supplementary material for: High-resolution crystal structure of arthropod Eiger TNF suggests a mode of receptor engagement and altered surface charge within endosomes
Source: Commun Biol. 2019 Aug 6;2:293. doi: 10.1038/s42003-019-0541-0 (PMC6684607; doi:10.1038/s42003-019-0541-0)
Supplement: Supplementary file 1 — Supplementary Figures [file 42003_2019_541_MOESM1_ESM.pdf]

Supplementary figure 1

Exon 1:
ATGAGGAGACCCTTCATAGCGGCCCATTTCCACGGCAACACATCTCATTTGAATAGTGCGATACATGACCATTA
CAAAG|GTACATAA

Exon2:
TTTCCAG|GTAACGGTCTAGTGCGCGTGTCGCATGACGCTCCTCACGACGTGTGGTACCCTGCACCCTGGACC
GTGGCGTCTCCACATCCCCGGCCTACCCTCACTCGCACCGGACACGTGCATGTACACCACACTGGTGTTTACTT
AGTCTATGTACAG|GTAAGA

Exon 3:
CAG|ATTTACTACCTGGACAGCCACGACACTATCTCCTGGGTGCTACACCGCACCAATGCCGACATTGAGGGT
CGGGAGACACTCCTGCAGTGTGCTCAGTCATCATACTCGACTGAACCCATCGACAAGCCGAACTCTTGCTTCT
CAGCAGCTGCCTTATTCCTGAAGGCTGGTGACAGGCTAGCAGTGAGGAATACGGCAGGAGACAGGCACTCC
TTGATGCAACCAGAGAAGAGTTTCATTGGGCTGGTAAAGCTGGCTGATGCCGAAGATCCTACCCAAGAGCTG
TAG

Supplementary figure 1 Genomic sequences for *S.frugiperda* Eiger. Bases highlighted red mark stop codons and bases highlighted in light blue mark proposed splice sites. Underlined bases encode for residues in the protein sequence.

Supplementary figure 2

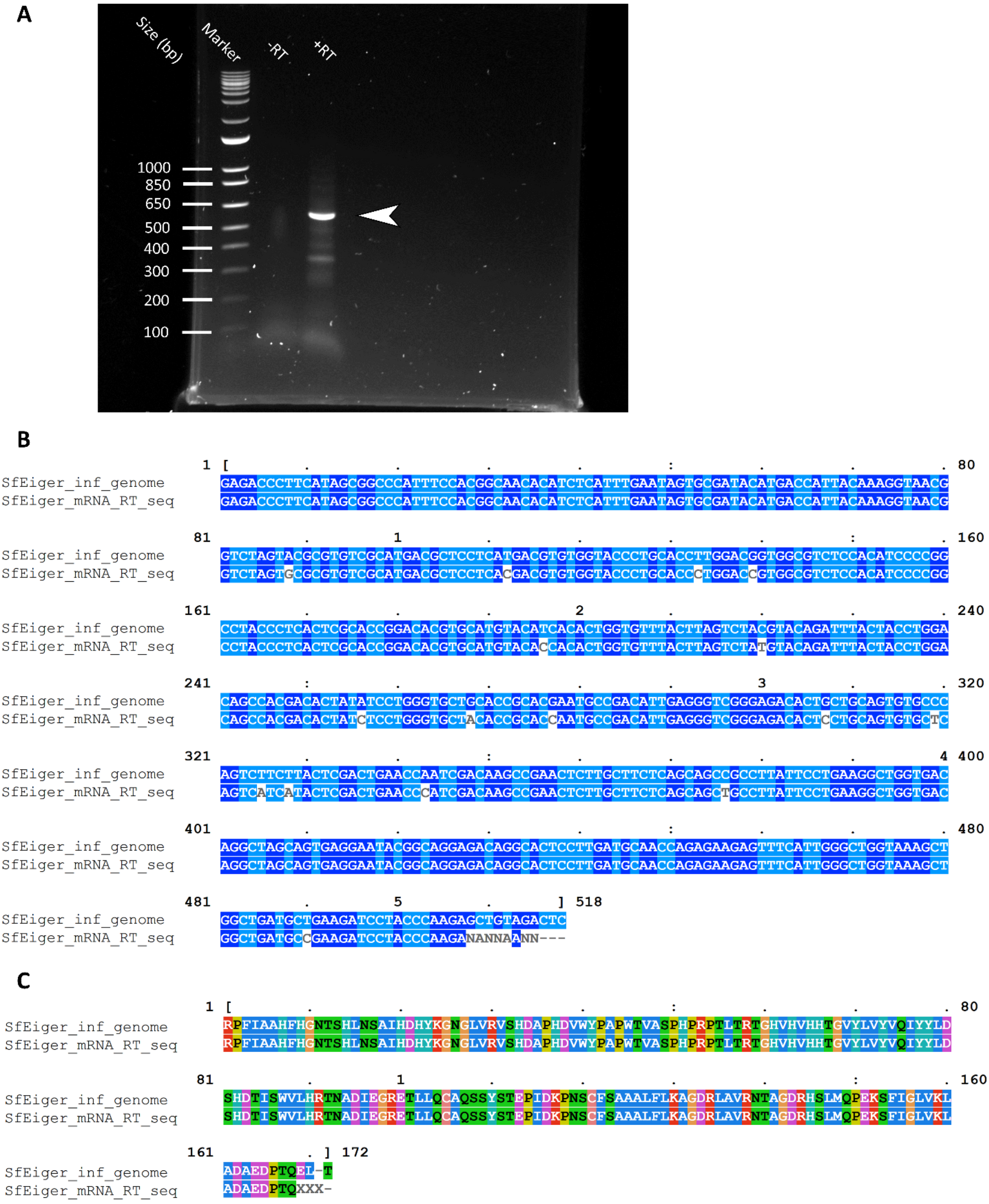

Supplementary figure 2: RT-PCR of SfEiger. (**A**) 1% agarose gel of the RT-PCR. The band of SfEiger is indicated with a white arrow. (**B**) Nucleotide sequence alignment of SfEiger. Top sequence is inferred from the *S. frugiperda* genome, bottom sequence is the result of the sequencing of the RT-PCR product. (**C**) Sequence alignment of the corresponding translated amino acid sequences, coloured according to the clustal scheme.

Supplementary figure 3

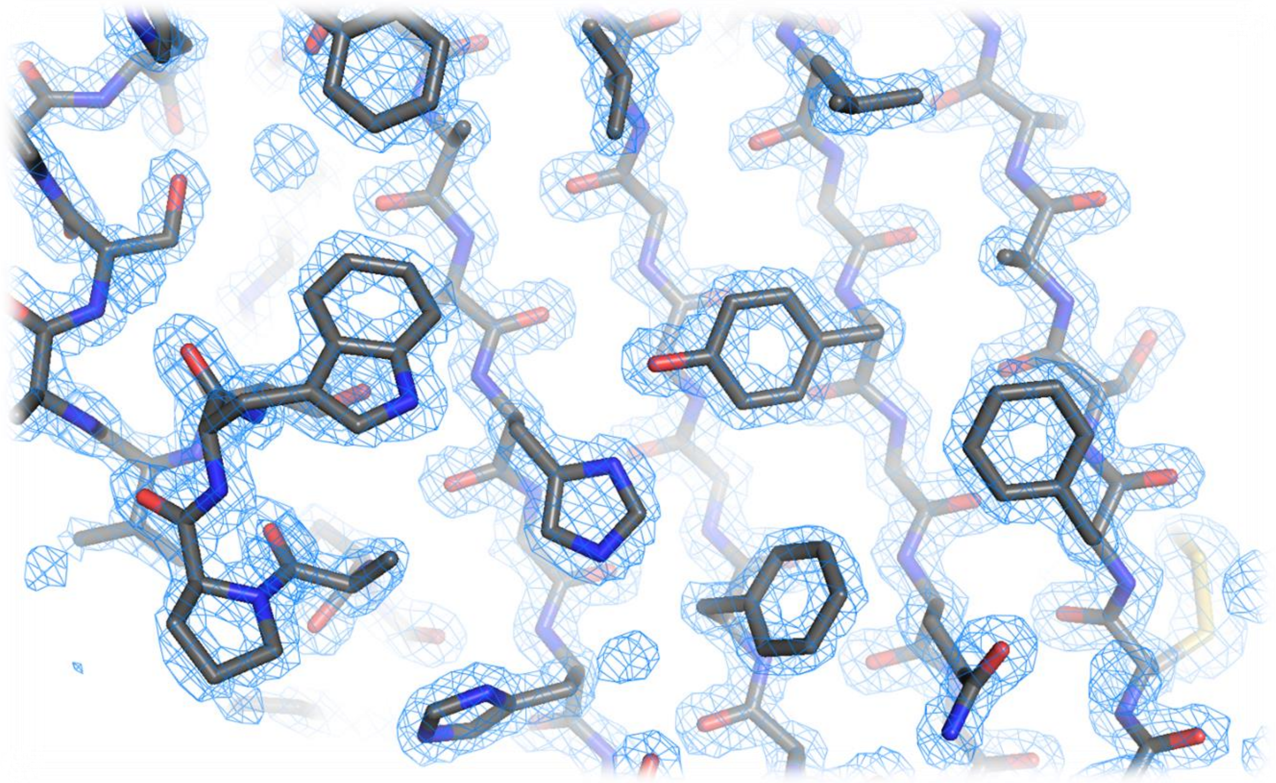

Supplementary figure 3 Representative density from the 2Fo-Fc electron density map contoured at 2 σ after the final round of refinement.

Supplementary figure 4

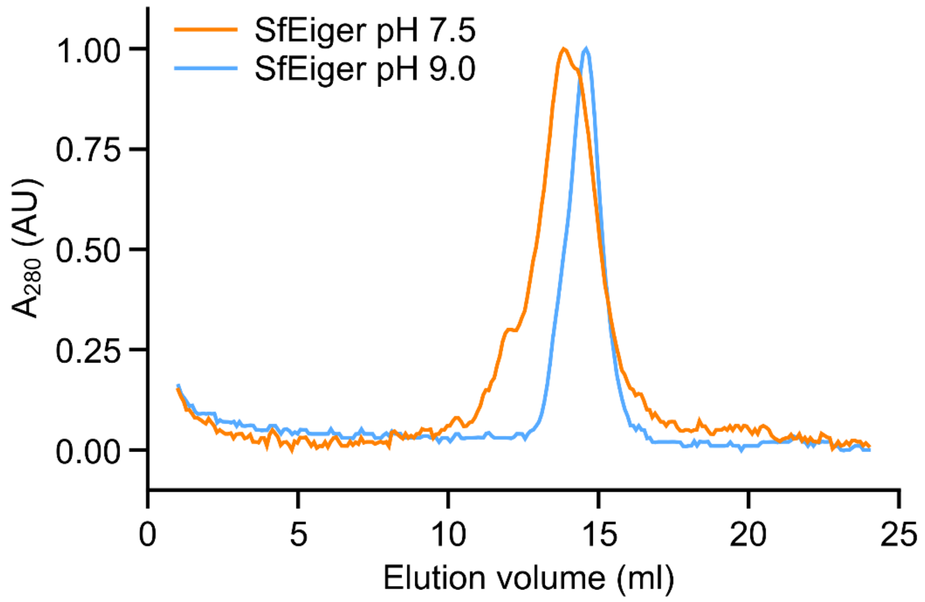

Supplementary figure 4 Size Exclusion Chromatography (SEC) profiles of SfEiger. The orange and blue traces refer to SEC runs in buffers with pH 7.5 and pH 9.0, respectively.

Supplementary figure 5

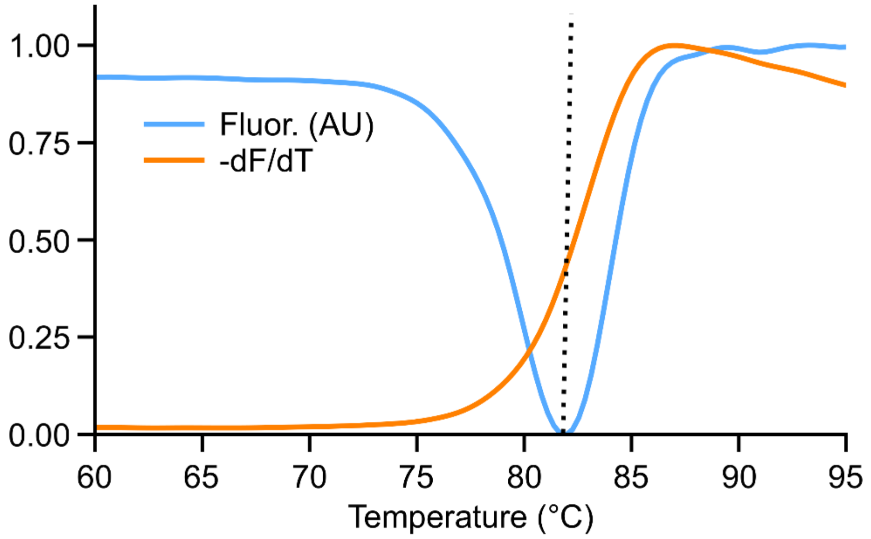

Supplementary figure 5 Differential scanning fluorescence analysis of SfEiger. The orange trace indicates the normalized fluorescence signal, while the blue trace indicates the derivative of the signal as function of the temperature. The minimum of the derivative plot indicates an approximate melting temperature of 82°C.

Supplementary Table 1: List of structures used in the generation of the phylogenetic tree of Figure 3C

Name	PDB Code
Human Lymphotoxin alpha	4mxv
Human Tumor necrosis factor	1tnf
Human Lymphotoxin beta	4mxw
Human OX40 ligand	2hev
Human CD40 ligand	3lkj
Human Fas ligand	5l19
Human CD137 ligand	6bwv
TNF-related apoptosis-inducing ligand	1dg6
Human Receptor activator of nuclear factor kappa-B ligand	3urf
Human TNF-related weak inducer of apoptosis	4ht1
Human A proliferation-inducing ligand	4zch
Human B-cell activating factor	5y9j
Human LIGHT	4en0
Human Vascular endothelial growth inhibitor	2rjl
Human TNF superfamily member 18	2r32
Human Ectodysplasin A	1rj7
