# Supplementary material for: High-resolution crystal structure of arthropod Eiger TNF suggests a mode of receptor engagement and altered surface charge within endosomes
Source: Commun Biol. 2019 Aug 6;2:293. doi: 10.1038/s42003-019-0541-0 (PMC6684607; doi:10.1038/s42003-019-0541-0)
Supplement: Supplementary file 2 — Description of Additional Supplementary Materials [file 42003_2019_541_MOESM2_ESM.docx]

**Description of Additional Supplementary Information**

Supplementary Data 1 contains raw absorbance values (AU) at 280nm vs elution volumes (ml) for size exclusion chromatography runs of recombinantly expressed SfEiger and a gel filtration protein standard (Bio-Rad). A Superdex 200 Increase 10/300GL column (GE Healthcare) was used.
