# Supplementary material for: High-resolution crystal structure of arthropod Eiger TNF suggests a mode of receptor engagement and altered surface charge within endosomes
Source: Commun Biol. 2019 Aug 6;2:293. doi: 10.1038/s42003-019-0541-0 (PMC6684607; doi:10.1038/s42003-019-0541-0)
Supplement: Supplementary file 5 — Reporting Summary [file 42003_2019_541_MOESM5_ESM.pdf]

nature research

Corresponding author(s): Max Renner

Last updated by author(s): Jul 1, 2019

Reporting Summary

Nature Research wishes to improve the reproducibility of the work that we publish. This form provides structure for consistency and transparency in reporting. For further information on Nature Research policies, see Authors & Referees and the Editorial Policy Checklist .

Statistics

For all statistical analyses, confirm that the following items are present in the figure legend, table legend, main text, or Methods section.

n/a	Confirmed	
☒	☐	The exact sample size (n) for each experimental group/condition, given as a discrete number and unit of measurement
☒	☐	A statement on whether measurements were taken from distinct samples or whether the same sample was measured repeatedly
☒	☐	The statistical test(s) used AND whether they are one- or two-sided<br>*Only common tests should be described solely by name; describe more complex techniques in the Methods section.*
☒	☐	A description of all covariates tested
☒	☐	A description of any assumptions or corrections, such as tests of normality and adjustment for multiple comparisons
☒	☐	A full description of the statistical parameters including central tendency (e.g. means) or other basic estimates (e.g. regression coefficient) AND variation (e.g. standard deviation) or associated estimates of uncertainty (e.g. confidence intervals)
☒	☐	For null hypothesis testing, the test statistic (e.g. F, t, r) with confidence intervals, effect sizes, degrees of freedom and P value noted<br>*Give P values as exact values whenever suitable.*
☒	☐	For Bayesian analysis, information on the choice of priors and Markov chain Monte Carlo settings
☒	☐	For hierarchical and complex designs, identification of the appropriate level for tests and full reporting of outcomes
☒	☐	Estimates of effect sizes (e.g. Cohen's d, Pearson's r), indicating how they were calculated

Our web collection on statistics for biologists contains articles on many of the points above.

Software and code

Policy information about availability of computer code

Data collection	XIA2 0.5.580-g8b4a78df-dials-1.10
Data analysis	PHASER ver 2.8.2, SHELXE version 2018/2, COOT ver. 0.8.9.1, REFMAC from CCP4 suite ver. 7.0.068 , Phenix ver. dev-3386, BLAST ver 2.8.0, NCBI Genome Workbench ver. 2.12.10, PHYLIP ver. 3.2, PYMOL ver. 1.8.6.0, Jalview ver. 2.10.5, DALI lite ver.4, PISA at PDBe ver.1.52, MolProbity ver. 4.4

For manuscripts utilizing custom algorithms or software that are central to the research but not yet described in published literature, software must be made available to editors/reviewers. We strongly encourage code deposition in a community repository (e.g. GitHub). See the Nature Research guidelines for submitting code & software for further information.

Data

Policy information about availability of data

All manuscripts must include a data availability statement. This statement should provide the following information, where applicable:

- Accession codes, unique identifiers, or web links for publicly available datasets
- A list of figures that have associated raw data
- A description of any restrictions on data availability

Coordinates and structure factors have been deposited in the Protein Data Bank (rcsb.org) with accession code 6I50

Field-specific reporting

Please select the one below that is the best fit for your research. If you are not sure, read the appropriate sections before making your selection.

Life sciences study design

All studies must disclose on these points even when the disclosure is negative.

Sample size	No ex-vivo experiments were performed. This study makes use of purely in-vitro data
Data exclusions	No data was excluded
Replication	Crystal growth was not replicated as sufficient data with high redundancy could be collected from one crystal
Randomization	We were not doing randomised trials. This is a structural analysis study
Blinding	This is a structural analysis study. No in-vivo studies were carried out

Reporting for specific materials, systems and methods

We require information from authors about some types of materials, experimental systems and methods used in many studies. Here, indicate whether each material, system or method listed is relevant to your study. If you are not sure if a list item applies to your research, read the appropriate section before selecting a response.

Materials & experimental systems

n/a	Involved in the study
☒	☐ Antibodies
☐	☒ Eukaryotic cell lines
☒	☐ Palaeontology
☒	☐ Animals and other organisms
☒	☐ Human research participants
☒	☐ Clinical data

Methods

n/a	Involved in the study
☒	☐ ChIP-seq
☒	☐ Flow cytometry
☒	☐ MRI-based neuroimaging

Eukaryotic cell lines

Policy information about cell lines

Cell line source(s)	SF9 cells from ThermoFisher cat.n. 11496015
Authentication	None of the cell lines were authenticated
Mycoplasma contamination	Cells were not tested for mycoplasma contamination
Commonly misidentified lines (See ICLAC register)	No commonly misidentified cell lines were used
